# Public Grants for Voluntary Hospitals

**Published:** 1910-11-19

**Authors:** 


### PUBLIC GRANTS FOR VOLUNTARY HOSPITALS.

### II.—IMPEDIMENTS IN ENGLAND.

The American system of subsidising private hospitals by the State or by the municipality has never taken root in this country. The line of demarcation has never been clearly fixed between the patient who can pay, if only a little, and the patient who cannot afford to pay at all. And hence the free beds which in America are a mere fraction of the whole become the *raison d'être* of the British hospital, even although those patients who are absolutely destitute are expected to resort to another class of institution.

#### Poor Law Institutions.

It is manifest to all who carefully digest the evidence brought before the Poor Law Commission that defects in the Poor Law are responsible for the want of co-operation between the parochial authorities and the voluntary hospitals. The moment for including the hospitals in a comprehensive scheme of assistance to the sick was when the country was being covered with workhouses, or again later on when separate provision for the sick was found essential. Hospitals ought then to have been invited to receive such cases as in their judgment were suitable ones and should have been paid for them *per capita* on a fixed ratio. By this means an immense stimulus would have been afforded to voluntary hospital work all over the country, for in all other countries the promise of public support has had a very happy effect in encouraging the foundation of charitable institutions. The ratepayers would have been relieved from the onus of attempting work which in the majority of instances they are wholly unfitted to undertake, and the zeal and kindly feeling in the community, so persistently set at nought by Poor Law influences, might have been secured for the permanently disabled as well as for the acutely ill. The opportunity was thrown away. Poor Law authorities have everywhere been compelled to ignore the existence of voluntary hospitals, and co-operation is rendered impracticable by the withering effects of the Poor Law administration. The hospitals do not care to have their inmates classed as "paupers." They dislike the sense of contamination which painfully clings to the mere suggestion of rate aid, and they have struggled on in the teeth of every possible discouragement from local authorities to keep ideals of medical science and nursing art alive through the far-sighted benevolence of a scanty minority of the population.

The impasse created by Poor Law incompetence is in many places very serious. The Poor Law institutions are, with few exceptions, lacking in the means to provide skilled surgical treatment for their patients. Recent forms of electric treatment have never penetrated to the Poor Law wards. Nursing is carried out under grave drawbacks and even dangers. The complete revolution accomplished in the treatment of tuberculosis is only slowly taking effect in workhouse sick wards, and yet in almost every locality the hospitals are holding out the torch, and with a little encouragement from public authorities could do all that is now being left undone. Nothing but the break-up of the present bad system, with its old evil traditions stultifying every effort at reform, will bring the hospitals into their right place as subsidised institutions of high national importance. At present grants to voluntary hospitals from the rates are rendered impracticable by the existence of separate rate-supported institutions. The sums collected for the burial of pauper patients and, under certain ill-defined conditions, for the maintenance of a few pauper patients represent all that the hospitals get out of the rates. The London Hospital in 1908 received only £95 from the Guardians—a sum ridiculously out of proportion to the extent of the work performed for destitute persons.

#### School Clinics.

The education authorities show a strong disposition to make use of the hospitals in the development of medical work among the children. Whether ultimately school clinics will be established in large centres under the control of the Board of Education or whether the hospitals will be encouraged by adequate payments to make provision for the required attendances remains a much-disputed question. Believing, as we do, that co-operation between the voluntary hospitals and the public authorities is the vital need of the day in the relief of the sick, we should welcome any well-thought-out system through which hospitals might undertake this branch of public work. The danger, however, that more eleemosynary work might be thrust upon a profession already heavily handicapped in this direction needs, however, to be guarded against, and the familiar disposition of municipal authorities to get something for nothing if they can has to be combated. The position of the doctor is one of the salient features in the problem of making public grants to medical charities. It is not a little unreasonable that as a ratepayer he should be mulcted in order to lengthen a little farther that chain of free medical relief which hinders him from earning his living.

### New York *versus* London.

Thus there would appear to be impediments and objections to almost any form of State or parish subsidy for the voluntary hospitals. We propose to show how some of these difficulties may be overcome, but meanwhile it is interesting to observe how the zealous altruism of English people has achieved for the London hospitals by private initiative the benefits procured for the hospitals in New York by the State Board of Charities. This Board distributes every year to private hospitals subsidies calculated on a *per capita* basis. It lays down salutary rules to be observed by those hospitals participating in the grants, and is thus able to exercise a strong influence in favour of economy and efficiency, as well as to afford a measure of support without which many of the private hospitals could never carry on their work. In the year 1906 the amount of public money distributed to private hospitals in New York through the State Board of Charities amounted to nearly £140,000. The amounts distributed among the voluntary hospitals of London during the same year from the King's Fund and the Hospital Sunday Fund together amounted to £173,075. Thus the voluntary hospitals have obtained from these central organisations £33,000 more than was voted by so wealthy a city as New York for the support of private institutions for the care of the sick. It is certainly a striking witness to the power of charity, and should give food for reflection to those who would see all the hospitals claimants for State support. It is worthy of note, too, that the rules laid down for the guidance of the distribution committees of the two funds enable them to promote the higher interests of hospital work while leaving individual corporations entirely free to develop along their own lines.

